# Implementing High-Intensity Interval Training in Physical Education: Effects on Adolescents’ Exercise Motivation

**DOI:** 10.3390/bs15040501

**Published:** 2025-04-09

**Authors:** Petar Mitić, Rade Jovanović, Nenad Stojiljković, Nebojša Trajković, Mihai Olanescu, Adrian Suciu, Danut Popa, Miruna Peris

**Affiliations:** 1 Department of Theoretical-Methodological Subjects, Faculty of Sport and Physical Education, University of Niš, 18000 Niš, Serbia; mitic.petar@gmail.com (P.M.); radejovanovic75@gmail.com (R.J.); snesadif@yahoo.com (N.S.); nele_trajce@yahoo.com (N.T.); 2 Physical Education Department, Faculty of Medicine, University of Niš, 18000 Niš, Serbia; 3 Faculty of Automotive, Mechatronics and Mechanical Engineering, Technical University of Cluj-Napoca, 400641 Cluj-Napoca, Romania; adrian.suciu@mdm.utcluj.ro (A.S.); popa_iliuta@yahoo.com (D.P.); 4 Faculty Industrial Engineering, Robotics and Production Management, Technical University of Cluj-Napoca, 400641 Cluj-Napoca, Romania; miruna.peris@muri.utcluj.ro

**Keywords:** exercise, motivational factors, Tabata, physical education

## Abstract

(1) Background: The aim of this study was to determine the effects of high-intensity interval training (HIIT) implemented in physical education classes on adolescents’ motivation for exercise. (2) Methods: This study involved 60 male adolescents (16.23 ± 0.6 years) recruited from a local high school who were randomly assigned to either the HIIT group or the control group (CG). The Exercise Motivation Inventory-2 (EMI-2) questionnaire was used to assess how participation in a HIIT program influenced the motivation to exercise among adolescents. The experimental program consisted of a 12-week HIIT program, integrated into the preparatory part of physical education classes. Sessions were held twice weekly, with each session lasting ~10 min. The HIIT group performed the Tabata protocol, which consisted of two 4 min sequences of eight different high-intensity exercises (burpees, split jumps, jumping jacks, push-ups, wall ball, crunches, frog jumps, and Russian twists), each lasting 20 s with 10 s rest intervals, separated by a 1 min recovery period. The control group conducted the traditional moderate-intensity warm-up exercises for the same total duration. After the warm-up protocols, both groups continued with the same physical education classes, which included a variety of instructional and moderate-intensity activities. (3) Results: A significant time × group interaction was observed for social recognition (*p* = 0.04; partial eta squared η^2^_p_ = 0.079, medium effect), indicating that HIIT had a superior effect compared to CG. Affiliation also showed a significant improvement in the HIIT group (*p* = 0.02), while no significant changes were noted in the CG (*p* = 0.35). Similarly, competition significantly increased in the HIIT group (*p* = 0.02), whereas no significant differences were found in the CG (*p* = 0.74). For all other motivational factors, no significant effects of time or group factors were found (*p* > 0.05). (4) Conclusions: This study highlights the positive effects of school-based HIIT on male adolescents’ motivation, particularly in the area of social recognition.

## 1. Introduction

Regular physical exercise is essential for the physical and mental health of children and adolescents (Biddle et al., 2019). It is well documented that exercise interventions can improve cardiorespiratory fitness, muscular strength, and mental health in adolescents (Rodriguez-Ayllon et al., 2019; Zhou et al., 2024). Specifically, Zhou et al. (2024) found that exercise interventions of varying intensities, including both high-intensity and moderate-intensity activities, significantly improved key aspects of health-related physical fitness in adolescents, including cardiovascular endurance, muscular strength, and body composition. Despite these clear health benefits, research indicates that adolescent physical activity (PA) levels remain below the recommended guidelines, potentially increasing the risk of anxiety, depression, and diminished cognitive functioning (McLure et al., 2009; Logan et al., 2014). Specifically, research indicates that 81% of adolescents between the ages of 11 and 17 worldwide are not meeting the recommended levels of physical activity (Rhodes et al., 2017). Therefore, it is imperative to develop strategies that enhance youth engagement in effective, enjoyable, and practical physical activities.

Motivation plays a crucial role in determining adolescents’ engagement in physical exercise, influencing both the initiation and maintenance of regular activity (Gillison et al., 2006). The aforementioned authors stated that higher motivation toward physical activity is associated with improved physical health and well-being, and understanding the factors that drive exercise behavior is essential for promoting long-term fitness habits in youth. To assess these motivations, researchers have developed several tools, one of which is the Exercise Motivations Inventory-2 (EMI-2), a validated and reliable instrument widely used to measure various exercise motives (Kim & Cho, 2022). The EMI-2 has been adapted for different populations, including adolescents, and has demonstrated good construct validity and internal consistency (Ivanović & Ivanović, 2018). In terms of the most significant exercise motives for adolescents, enjoyment, appearance, and health-related factors such as weight control and stress management are frequently reported as the primary drivers (Navarro et al., 2021; Kim & Cho, 2022). These motivations reflect the importance of both intrinsic and extrinsic factors in encouraging physical activity among young people.

Adolescents’ motivation toward physical exercise is a complex construct influenced by multiple factors, including individual characteristics such as age, gender, and personal goals (Navarro et al., 2021). The EMI-2, as mentioned above, is a validated tool (Kim & Cho, 2022) that includes several subscales that assess motives such as health, enjoyment, appearance, and social recognition, which have been found to vary by demographic factors. For example, studies have shown that male adolescents tend to be more motivated by competition and strength goals, while female adolescents prioritize weight control and health-related motives (Portela-Pino et al., 2020; Navarro et al., 2021). These findings underscore the importance of understanding the specific motivations within subgroups, as they reflect the distinct psychological needs and behaviors that drive exercise engagement.

School-based interventions have been identified as effective strategies for improving motivation toward physical activity among adolescents, providing a structured environment to address barriers and enhance intrinsic motivation (Demetriou et al., 2019). Research has consistently shown that such interventions can positively influence motivation by fostering enjoyment, competence, and autonomy in physical activity settings (Demetriou et al., 2019; Sevil-Serrano et al., 2022). Programs like the PESSOA intervention have demonstrated that increased social support can lead to higher exercise motivation, which in turn improves physical activity levels and overall quality of life (Quaresma et al., 2014). Similarly, interventions focusing on structured walking programs or active participation in physical education (PE) classes have been found to shift students’ motivation towards more intrinsic forms, emphasizing personal enjoyment and long-term adherence to physical activity (Brustio et al., 2018; Barkoukis et al., 2021). Furthermore, interventions that incorporate motivational strategies, such as goal setting and peer encouragement, can enhance adolescents’ willingness to engage in physical activity beyond the school environment (Barkoukis et al., 2021). These findings underscore the importance of designing school-based exercise programs that not only improve physical fitness but also cultivate a motivational climate that supports sustained physical activity participation among adolescents.

A significant barrier to exercise among youth is a lack of time (Atakan et al., 2021). In this context, high-intensity interval training (HIIT) emerges as a promising alternative, as it provides substantial health benefits within a shorter duration compared to traditional moderate-intensity exercise. Researchers have demonstrated that HIIT can positively impact adolescent mental health, including cognitive function improvement, reductions in anxiety and depressive symptoms, and increased motivation for exercise (Costigan et al., 2016; Alves et al., 2021). Given that young individuals spend a substantial portion of their day at school, educational institutions serve as an ideal setting for promoting HIIT within physical education curricula. The most recent study suggests that students who are offered choice and variety in HIIT sessions exhibit higher motivation to participate in physical activities (Eather et al., 2025). Additionally, this study highlighted that motivational factors such as affiliation and social recognition were more pronounced in adolescents who had greater control over their workouts, reinforcing the importance of individualized approaches in school-based HIIT programs.

Motivation plays a crucial role in exercise adherence, and HIIT programs have been shown to significantly influence motivation levels. Heinrich et al. (2014) and Fisher et al. (2017) found that individuals participating in HIIT programs reported higher motivation levels, as measured by the EMI-2 questionnaire. Specifically, their studies indicated that HIIT participants exhibited greater motivation compared to those engaged in other exercise modalities, such as strength training. This heightened motivation is thought to be one of the key benefits of HIIT programs, contributing to higher engagement and adherence in exercise routines.

Given the need to develop strategies to enhance youth engagement in physical activity, the rationale for adopting the HIIT approach in this study lies in its practical advantages and effectiveness. HIIT is particularly suitable for physical education (PE) classes because it is easy to implement, requiring minimal equipment and space. The short duration of HIIT sessions, typically lasting between 10 and 20 min, makes it feasible to incorporate into the existing structure of PE classes without requiring additional time commitments from students. Moreover, HIIT has been shown to be time-efficient, delivering significant improvements in cardiovascular fitness, muscular strength, and overall physical health within a relatively short period. This makes it an ideal approach to boost engagement and fitness levels among adolescents, particularly in the context of structured school programs where time constraints often limit the opportunity for longer, more traditional exercise regimens.

Although a considerable body of research has examined the effects of HIIT on the physical and mental health of youth, there remains a research gap concerning the role of motivation in exercise participation, which is a critical determinant of long-term PA engagement and behavioral change. Understanding the motivational mechanisms underlying HIIT participation among adolescents may facilitate the development of more effective programs that encourage sustained involvement in physical activities. Therefore, the aim of this study was to determine the effects of HIIT implemented in physical education classes on male adolescents’ motivation for exercise. It was hypothesized that adolescents participating in school-based HIIT will exhibit higher levels of motivation for exercise compared to those engaged in traditional physical education activities.

## 2. Materials and Methods

### 2.1. Participants

This study involved 60 male adolescents (16.23 ± 0.6 years) recruited from a local high school. Basic descriptive characteristics for both groups can be seen in Table 1. Participants were randomly assigned to either the HIIT group (30 participants) or the control group (30 participants), ensuring that the two groups were comparable in terms of age, gender, and physical fitness levels. The random assignment was performed using a computer-generated randomization list.

Inclusion criteria for the study included adolescents who were generally healthy, with no history of cardiovascular, musculoskeletal, or neurological disorders, and who had not participated in any structured exercise program for the past six months. Exclusion criteria included individuals with chronic medical conditions, injuries that could interfere with exercise, or those who had previously participated in a high-intensity exercise regimen.

All participants successfully completed the 12-week intervention, with no dropouts due to personal, health-related, or other reasons. Attendance records indicated that all participants attended at least 90% of the scheduled sessions. Minor absences due to short-term illnesses (e.g., colds) were documented; however, no participant was absent for a duration that met the criteria for dropout.

Before the study commenced, written consent was obtained from the participants’ parents and the grammar school principal, and the participants had the right to withdraw from the study at any point. Additionally, each parent provided written authorization for their child’s voluntary participation. Both the children and their parents were fully informed about the study’s significance and potential benefits. The research received approval from the Ethical Committee of the Faculty of Sport and Physical Education. This study was conducted in accordance with the Declaration of Helsinki and was carried out as part of a doctoral dissertation, officially approved by the University of Niš, Serbia (decision No. 8/18-01-007/23-031, approval date 6 November 2023).

### 2.2. Procedures

The Exercise Motivation Inventory-2 (EMI-2), developed by Markland and Ingledew (1997), is a comprehensive tool used to assess the diverse range of motivations individuals have for engaging in physical activity. The EMI-2 consists of 51 items, with responses rated on a 6-point Likert scale ranging from 0 (does not apply to me at all) to 5 (applies to me very much). Higher scores indicate a stronger motivation to exercise. The scale is divided into 14 subscales that represent different motivational factors for exercise: enjoyment, challenge, revitalization, stress management, affiliation, social recognition, competition, health pressures, ill-health avoidance, positive health, appearance, weight management, strength and endurance, and nimbleness. Each subscale is calculated by averaging 3 to 4 items, based on the EMI-2 scale’s scoring guidelines.

The EMI-2 has been shown to be a reliable and valid tool for assessing exercise motivation in a variety of populations, including both athletes and non-athletes, across different gender and age groups (Kim & Cho, 2022; Vuckovic & Duric, 2024). Ivanović and Ivanović (2018) reported high internal reliability for the EMI-2 subscales in Serbian adolescents, with Cronbach’s alpha coefficients ranging from 0.83 to 0.92. Moreover, Kim and Cho (2022) validated the EMI-2 among 325 college students. The authors have demonstrated that person ability and item difficulty were well matched, thereby supporting the construct validity of the EMI-2 in this population.

In the context of our research, the EMI-2 questionnaire was utilized to assess how participation in a HIIT program influenced the motivation to exercise among adolescents. The use of the EMI-2 allowed us to investigate changes in exercise motivation both at the baseline and after the 12-week HIIT intervention. Specifically, we aimed to explore whether HIIT, as a time-efficient and highly engaging form of exercise, would enhance motivation across various dimensions, such as enjoyment, revitalization, strength and endurance, and health-related goals. By measuring motivational variations, we sought to understand how HIIT could potentially foster greater long-term commitment to physical activity, which could contribute to improvements in physical fitness and overall health in adolescents.

### 2.3. Exercise Intervention

The HIIT protocol was designed to include high-intensity interval training exercises, with a focus on improving overall fitness and cardiovascular health. Throughout the HIIT sessions, participants’ heart rates were continuously tracked using wrist-worn monitors (Garmin Forerunner 245; Garmin Ltd., New Taipei City, Taiwan) to ensure that the participants exercised within the target intensity zone. These devices delivered real-time data, allowing instructors to oversee each session and ensure compliance with the intended heart rate zone. The intensity was set at 80–90% HRmax. If a participant’s heart rate dropped below 80% of their maximum, verbal prompts were provided to encourage greater exertion. On the other hand, if their heart rate exceeded 90% of the maximum, they were advised to reduce movement intensity or take short breaks to return to the target zone. This approach maintained a consistent workout intensity while prioritizing both safety and the effectiveness of the program.

The experimental segment of this study involved a 12-week HIIT program tailored for high school students, integrated into the preparatory part of physical education classes. Sessions were held twice weekly, with each session lasting approximately 10 min. To ensure comparability between groups, all participants engaged in a 5 min standardized warm-up routine. Following this, the HIIT group performed the Tabata protocol, which consisted of two 4 min sequences of eight different high-intensity exercises (burpees, split jumps, jumping jacks, push-ups, wall ball, crunches, frog jumps, and Russian twists), each lasting 20 s with 10 s rest intervals, separated by a 1 min recovery period.

In contrast, the control group continued with traditional moderate-intensity warm-up exercises for the same total duration. The traditional warm-up routine included a combination of low-intensity running and dynamic movements designed to increase heart rate and prepare the body for exercise. This warm-up began with light jogging, followed by dynamic exercises such as high knees, butt kicks, side slides, and skips, all aimed at improving the mobility and activation of key muscle groups. After these dynamic movements, the group engaged in both static and dynamic stretching exercises to further enhance flexibility and muscle readiness for the physical activity that followed. After the warm-up protocols, both groups continued with the same physical education classes, which included a variety of instructional and moderate-intensity activities. By maintaining equal session durations and ensuring similar total exercise exposure, any observed differences in outcomes could be attributed to the specific exercise modality rather than differences in overall training volume.

### 2.4. Statistical Analysis

All statistical analyses were conducted using SPSS version 24 (IBM Corp., Armonk, NY, USA). The normality of the data distribution was assessed prior to the analysis. Baseline differences between the HIIT and control groups were examined using an independent samples *t*-test. To evaluate differences over time and between groups, a two-factor (group × time) analysis of variance (ANOVA) with repeated measures was performed. When a main effect was identified, Bonferroni’s post hoc test was conducted to assess pairwise differences when applicable. Additionally, a partial eta (η) squared was used for measuring the difference between groups (0.01 = small effect, 0.06 = medium effect, and 0.14 = large effect). The significance level was set at *p* < 0.05.

## 3. Results

**Figure 1:**
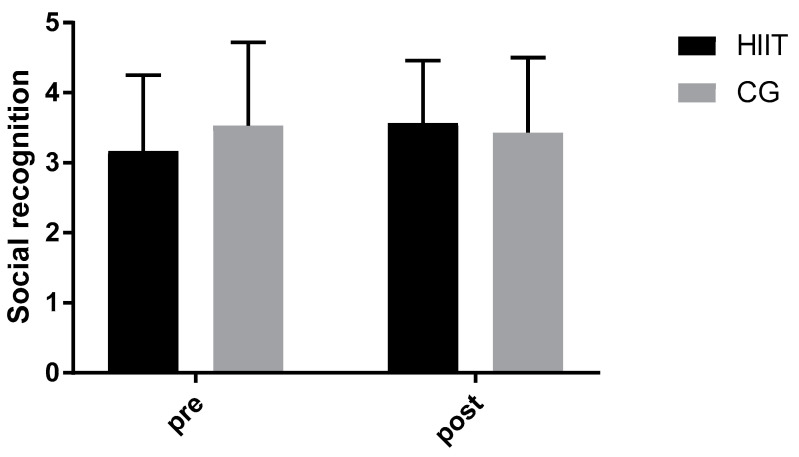
Significant time × group interaction for social recognition.

The results of the EMI-2 motivation questionnaire showed significant improvements in specific motivational factors in the HIIT group (Table 2). At the baseline, there were no statistically significant differences observed between the two groups (*p* > 0.05). A significant time × group interaction (Figure 1) was observed for social recognition (*p* = 0.04; partial eta squared η^2^_p_ = 0.079, medium effect), indicating that HIIT had a superior effect compared to CG. Social recognition significantly increased following HIIT (%difference = 11.86%, *p* = 0.02), whereas no significant change was observed in the CG (%difference = 2.87%, *p* = 0.92).

A significant main effect for time was found for affiliation (F_(1,58)_ = 6.39, η^2^_p_ = 0.05; *p* = 0.02) and competition (F_(1,58)_ = 5.78, η^2^_p_ = 0.04; *p* = 0.02) (Table 2). Affiliation showed a significant improvement in the HIIT group (%difference = 10.83%), while no significant changes were noted in the CG (%difference = 3.52%, *p* = 0.35). Similarly, competition significantly increased in the HIIT group (%difference = 12.14%), whereas no significant differences were found in the CG (%difference = 1.86%, *p* = 0.74). For all other motivational factors, no significant differences were found within or between groups (*p* > 0.05).

## 4. Discussion

The aim of this research was to explore the effects of school-based HIIT on motivation among adolescents, specifically examining aspects of social recognition, affiliation, and competition in comparison to traditional PE classes and a control group. The results of this study demonstrated that HIIT significantly improved social recognition, affiliation, and competition when compared to the control group and traditional PE classes. However, the only statistically significant difference between the groups was found in social recognition. This suggests that while HIIT may offer broader motivational benefits, its most profound impact was on how adolescents perceive their social recognition in the school setting.

The importance of exercise, particularly HIIT, in fostering motivation among adolescents is well documented in the recent literature. HIIT has been shown to be effective in increasing motivation and engagement in physical activity, with studies reporting improvements in both physical fitness and psychological outcomes (Aziz et al., 2024; Vuckovic & Duric, 2024; Mitić et al., 2024). Our findings align with these results, as the HIIT group exhibited greater motivation in social recognition and affiliation, which are key components of social interaction and peer relationships during adolescence (Eather et al., 2025). However, the fact that only social recognition showed a significant difference may be due to the specific dynamics of social development in adolescents, where peer recognition plays a critical role in motivation and participation in school-based activities. The sense of belonging and validation from peers might be more influenced by the social context created through HIIT than by other aspects such as affiliation or competition (Simonsen et al., 2024). Additionally, these findings may reflect the inherent structure of HIIT, which focuses on intense, individualized tasks that could foster a stronger sense of personal achievement and peer acknowledgment. Similarly to our results, previous school-based interventions have also reported positive effects on motivation for physical activity. Specifically, Brustio et al. (2018) found that participation in a walking intervention increased students’ motivation to engage in physical activity, while Barkoukis et al. (2021) noted that school-based programs enhanced motivation for extracurricular physical activity participation. Moreover, Sevil-Serrano et al. (2022) emphasized the role of structured physical education interventions in improving students’ motivation, reinforcing the importance of social factors in sustaining engagement. A notable comparison can be made with the study by Vuckovic and Duric (2024), which utilized the EMI-2 questionnaire to explore motivational differences across various exercise modalities. Their findings highlighted that social-related motives, including social recognition, varied based on fitness engagement patterns, supporting our results that HIIT can effectively enhance this aspect of motivation. Furthermore, studies such as those performed by Demetriou et al. (2019) and Quaresma et al. (2014) suggest that motivation shifts in response to intervention programs, with social factors playing a key mediating role. Collectively, these findings reinforce the idea that structured high-intensity training may be particularly beneficial for enhancing social recognition, a critical component of sustained exercise motivation.

The results of the current study align with previous findings regarding the motivational benefits of HIIT programs, particularly in terms of social recognition. Heinrich et al. (2014) and Fisher et al. (2017) highlighted that HIIT participants exhibited significantly higher motivation levels compared to other exercise modalities, which is consistent with our findings. Heinrich et al. (2014) compared high-intensity interval training (HIIT) with moderate-intensity training and found that HIIT participants reported significantly higher levels of exercise enjoyment, adherence, and exercise intentions. Specifically, this study showed that those in the HIIT group experienced greater increases in motivation to continue exercising, as well as a stronger intention to engage in physical activity in the future. In terms of the EMI-2, participants in the HIIT group scored higher on motivation related to enjoyment and exercise initiation, indicating that the intense, varied nature of HIIT sessions may be more appealing to individuals seeking excitement and novelty in their workouts. Fisher et al. (2017) examined motivational factors among CrossFit participants and compared them to individuals involved in other resistance exercise modalities. The study revealed that CrossFit participants, who engage in a form of HIIT, scored higher on motivation related to social recognition and competition, as measured by the EMI-2. This suggests that the community environment and competitive aspect of CrossFit contributed significantly to participants’ motivation to continue exercising, particularly in the areas of social recognition and achievement, which were more pronounced in HIIT groups compared to other exercise modalities. In our study, the short HIIT program improved social recognition motivation in adolescents, which is a critical factor for this age group as it influences their exercise behaviors and adherence. This suggests that HIIT not only enhances physical fitness but also fosters important psychosocial motivations, making it an effective tool for promoting exercise among adolescents.

The absence of significant differences in affiliation and competition as well as in other components of motivation may point to the complex and multifactorial nature of motivation, which could be influenced by various individual and social factors. The lack of significant differences in other motivation factors may be attributed to several factors. First, adolescents’ prior exercise experience could influence their initial levels of motivation, potentially masking the effects of the intervention. Adolescents with more experience in physical activity might not show as large a change in motivation as those with less experience. Social factors, such as peer influence, could also affect motivation levels, with some adolescents being more influenced by their social environment than the intervention itself. Finally, the duration and intensity of the intervention might not have been sufficient to induce changes in certain motivation subscales, suggesting that a longer or more varied program could be needed to impact these factors more significantly. Future studies should aim to explore these dimensions in greater detail, considering factors such as gender, age, and baseline fitness levels, which may further contribute to a deeper understanding of HIIT’s impact.

Despite the interesting findings, this study has several limitations. First, the sample was exclusively composed of boys, which restricts the generalizability of the results to both genders. Gender differences in motivation, as suggested by previous research (Leahy et al., 2024), could have influenced how HIIT impacted motivation across different social dimensions. Furthermore, the control group performed traditional warm-up exercises, but this study does not explore whether another form of exercise might yield different motivational effects. Other limitations include the reliance on self-reported measures of motivation, which may introduce biases, and the lack of a more comprehensive assessment of individual factors, which could influence the outcomes. To address the limitations of self-reported measures of motivation, future research could incorporate objective measures such as behavioral observations or fitness assessments, use multiple data sources (e.g., teacher or peer evaluations), and apply experience sampling methods to capture real-time motivation. Longitudinal designs with repeated measurements could help track changes over time. Additionally, it would be beneficial to include a more diverse sample, encompassing different age groups, cultural backgrounds, and varying levels of physical fitness, to better capture the broader effects of HIIT on adolescent motivation across different contexts.

## 5. Conclusions

This study highlights the positive effects of school-based HIIT on adolescent motivation, particularly in the area of social recognition. While HIIT significantly improved motivation in terms of social recognition, affiliation, and competition, only social recognition showed a statistically significant difference compared to the control group. These findings suggest that HIIT, with its individualized and high-energy nature, may foster a stronger sense of peer acknowledgment, which is a key motivator for adolescents. Given the growing importance of promoting physical activity among young people, especially in the school setting, HIIT presents a promising approach to enhancing motivation and engagement in physical activity.

## Figures and Tables

**Table 1:** Basic anthropometric parameters at baseline (pre) and after (post) the intervention program in high-intensity interval training group and control group.

HIIT				CG		
	Pre	Post	*p*-Value	Pre	Post	*p*-Value
Height (cm)	176.6 ± 8.4	176.8 ± 7.7	0.98	175.8 ± 5.8	176.1 ± 6.4	0.48
Body mass (kg)	67.2 ± 13.2	66.7 ± 12.4	0.88	69.1 ± 13.5	70.3 ± 13.2	0.75
BMI (kg/m^2^)	21.2 ± 3.5	21.3 ± 3.3	0.90	22.1 ± 3.4	22.5 ± 3.3	0.82

HIIT—high-intensity interval training group; CG—control group; BMI—body mass index.

**Table 2:** Exercise Motivation Inventory-2 scores at baseline (pre) and after (post) the intervention Program in high-intensity interval training group and control group.

	HIIT (n = 30)			CG (n = 30)		
Motivation Subscales	Pre	Post	*p*-Value	Pre	Post	*p*-Value
Stress management	3.68 ± 0.93	3.76 ± 0.91	0.42	3.70 ± 1.10	3.87 ± 0.88	0.49
Revitalization	4.07 ± 0.92	3.89 ± 0.96	0.57	3.98 ± 1.01	3.84 ± 0.98	0.28
Enjoyment	4.04 ± 0.97	3.94 ± 0.98	0.80	4.06 ± 1.12	4.13 ± 0.91	0.83
Challenge	3.92 ± 0.83	4.08 ± 0.90	0.12	4.11 ± 1.12	4.22 ± 0.81	0.76
Social recognition †	3.17 ± 1.08	3.57 ± 0.89	0.02 *	3.53 ± 1.19	3.43 ± 1.07	0.97
Affiliation	3.58 ± 1.17	3.99 ± 0.80	0.02 *	3.62 ± 1.14	3.75 ± 1.06	0.35
Competition	3.48 ± 1.19	3.93 ± 0.87	0.02 *	3.72 ± 1.18	3.77 ± 1.02	0.74
Health pressures	3.12 ± 0.96	3.17 ± 0.99	0.79	3.19 ± 1.23	3.41 ± 1.06	0.32
Ill-health avoidance	3.90 ± 1.07	3.89 ± 0.97	0.49	3.90 ± 1.16	3.71 ± 1.22	0.32
Positive health	4.23 ± 0.94	4.11 ± 0.87	0.55	4.23 ± 1.21	4.28 ± 0.90	0.88
Weight management	3.28 ± 0.80	3.42 ± 0.83	0.85	3.70 ± 1.10	3.73 ± 1.04	0.94
Appearance	3.53 ± 1.10	3.70 ± 0.97	0.19	3.68 ± 1.23	3.73 ± 1.00	0.95
Strength and endurance	4.38 ± 0.78	4.25 ± 0.72	0.20	4.22 ± 1.21	4.33 ± 0.71	1.00
Nimbleness	4.00 ± 0.88	4.03 ± 0.93	0.49	4.02 ± 1.12	4.04 ± 0.90	0.97

Data are expressed as mean ± SD; * significant time effect *p* < 0.05; † significant time × group interaction *p* < 0.05.

## Data Availability

The data presented in this study are available on request from the corresponding author.
